# Supplementary material for: Conditioned Generative Modeling of Molecular Glues: A Realistic AI Approach for Synthesizable Drug-like Molecules
Source: Biomolecules. 2025 Jun 10;15(6):849. doi: 10.3390/biom15060849 (PMC12190558; doi:10.3390/biom15060849)
Supplement: Supplementary file 1 [file biomolecules-15-00849-s001.zip › biomolecules-3642987-supplementary.pdf]

Supplemental material for “Conditioned Generative Modeling of Molecular Glues: A Realistic AI Approach for Synthesizable Drug-Like Molecules”

Naeyma N. Islam[1,‡], PhD; Thomas R. Caulfield, PhD[2,*]
[1] Department of Neuroscience, Mayo Clinic, Jacksonville FL
[2] CEO, Digital Ether Computing, Miami, FL

a)

```
mol = Chem.AddHs(mol)
AllChem.MMFFOptimizeMolecule(mol)
conf = mol.GetConformer()
```

b)

```
def get_torsional_angle(self, bond, mol, conf):
    """Compute torsional angle for a rotatable bond."""
    atom1 = bond.GetBeginAtom()
    atom2 = bond.GetEndAtom()
    neighbors1 = [n.GetIdx() for n in atom1.GetNeighbors() if n.GetIdx() !=
    atom2.GetIdx()]
    neighbors2 = [n.GetIdx() for n in atom2.GetNeighbors() if n.GetIdx() !=
    atom1.GetIdx()]

    if len(neighbors1) == 0 or len(neighbors2) == 0:
        return 0.0  # No valid torsional angle

    A, B = neighbors1[0], atom1.GetIdx()
    C, D = atom2.GetIdx(), neighbors2[0]

    return Chem.rdMolTransforms.GetDihedralDeg(conf, A, B, C, D)
```

Figure S1. Dihedral angles computed using RDKit’s GetDihedralDeg function. a) RDKit code snippet illustrating hydrogen addition (AddHs), molecular geometry optimization using the MMFF94 force field (MMFFOptimizeMolecule), and retrieval of the optimized conformer (GetConformer) for a given molecule. **b)** Custom Python function get_torsional_angle for computing the torsional (dihedral) angle across a rotatable bond. The function identifies neighboring atoms required for angle computation and uses *Chem.rdMolTransforms.GetDihedralDeg* to calculate the dihedral angle in degrees based on the conformer coordinates.

```
Model
JTNNVAE(
  (jtnn): JTNNEncoder(
    (embedding): Embedding(780, 450)
    (outputNN): Sequential(
      (0): Linear(in_features=900, out_features=450, bias=True)
      (1): ReLU()
    )
    (GRU): GraphGRU(
      (W_z): Linear(in_features=900, out_features=450, bias=True)
      (W_r): Linear(in_features=450, out_features=450, bias=False)
      (U_r): Linear(in_features=450, out_features=450, bias=True)
      (W_h): Linear(in_features=900, out_features=450, bias=True)
    )
  )
  (decoder): JTNNDecoder(
    (embedding): Embedding(780, 450)
    (W_z): Linear(in_features=900, out_features=450, bias=True)
    (U_r): Linear(in_features=450, out_features=450, bias=False)
    (W_r): Linear(in_features=450, out_features=450, bias=True)
    (W_h): Linear(in_features=900, out_features=450, bias=True)
    (W): Linear(in_features=478, out_features=450, bias=True)
    (U): Linear(in_features=478, out_features=450, bias=True)
    (U_i): Linear(in_features=900, out_features=450, bias=True)
    (W_o): Linear(in_features=450, out_features=212, bias=True)
    (U_o): Linear(in_features=450, out_features=1, bias=True)
    (pred_loss): CrossEntropyLoss()
    (stop_loss): BCEWithLogitsLoss()
  )
  (jtmpn): JTMPN(
    (W_i): Linear(in_features=40, out_features=450, bias=False)
    (W_h): Linear(in_features=450, out_features=450, bias=False)
    (W_o): Linear(in_features=485, out_features=450, bias=True)
  )
Binding Site Encoder (res_encoder) :
ResidueEncoder(
W_H1: Linear(in_features=450, out_features=450, bias=False),
W_H2: Linear(in_features=450, out_features=450, bias=False),
WO: Linear (in_features=450, out_features=450, bias=True))
  (mpn): MPN(
    (W_i): Linear(in_features=50, out_features=450, bias=False)
    (W_h): Linear(in_features=450, out_features=450, bias=False)
    (W_o): Linear(in_features=489, out_features=450, bias=True)
  )
  (A_assm): Linear(in_features=28, out_features=450,
bias=False)
  (assm_loss): CrossEntropyLoss()
  (T_mean): Linear(in_features=450, out_features=28, bias=True)
  (T_var): Linear(in_features=450, out_features=28, bias=True)
  (G_mean): Linear(in_features=450, out_features=28, bias=True)
  (G_var): Linear(in_features=450, out_features=28, bias=True)
)
```

Figure S2. Architecture of the JTNNVAE model for ligase-conditioned molecular glue generation. The model consists of a junction tree encoder (JTNNEncoder) that embeds

molecular graphs into a latent space, a graph-based message-passing network (GRUGraphGRU) to capture atom-level connectivity, and a junction tree decoder (JTNNDecoder) for reconstructing chemically valid clique structures. An additional binding site residue encoder processes E3 ligase binding site features (e.g., residue embeddings from ProtBERT), enabling the model to condition generation on specific ligase contexts. The MPN encoder integrates atomic features, while the atom assembly module combines latent vectors from the junction tree and residue encoder to assemble complete molecules. Finally, the model outputs ligase-specific compounds via three linear transformations (T_real, T_mean, and G_var) that capture the conditional generation dynamics. This architecture enables the targeted design of molecular glue compounds tailored to specific E3 ligase binding environments.

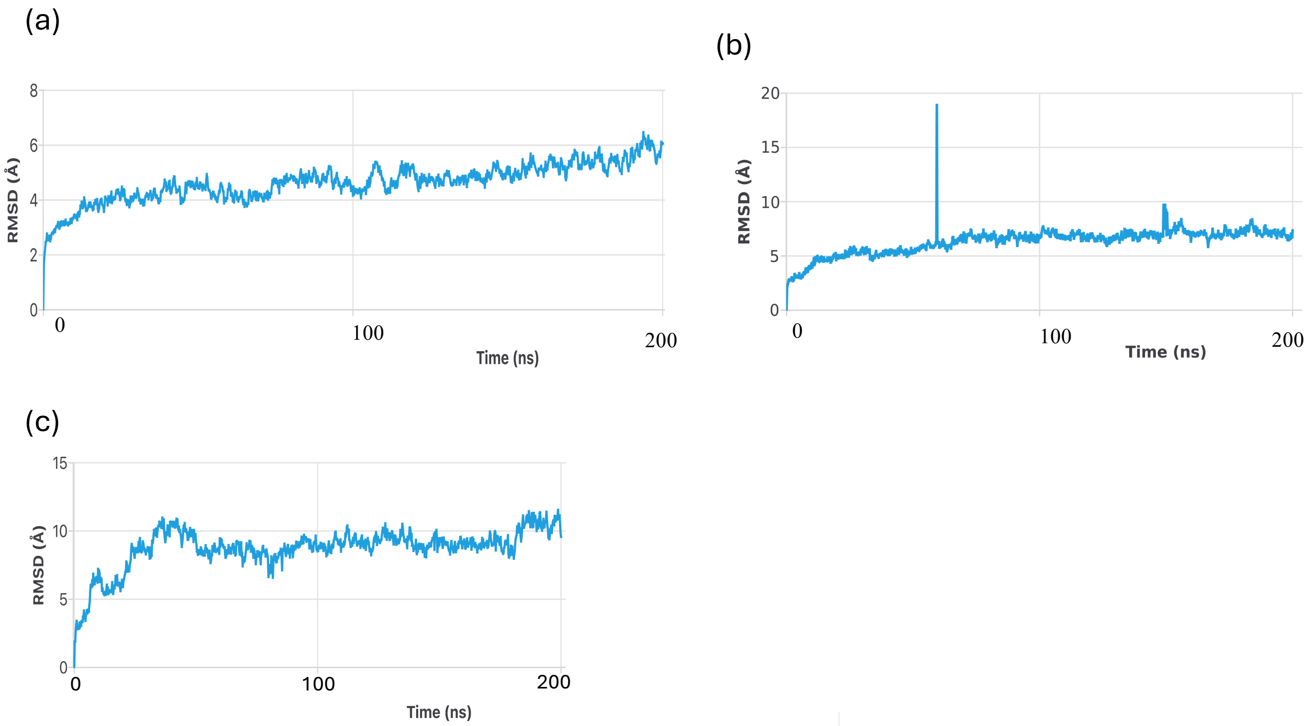

Figure S3. Root-mean-square deviation (RMSD) plots for ternary complexes of Aβ42, E3 ligases, and top-ranked ligands over 200 ns molecular dynamics simulations.
(a) VHL–ligand–Aβ42 complex, (b) MDM2–ligand–Aβ42 complex, and (c) CRBN–ligand–Aβ42 complex. RMSD values (in Å) were calculated for the entire ternary complex to evaluate structural stability over time. The relatively stable trajectories across all panels indicate that the ternary assemblies remained conformationally stable throughout the simulation period, supporting the feasibility of molecular glue–mediated complex formation.

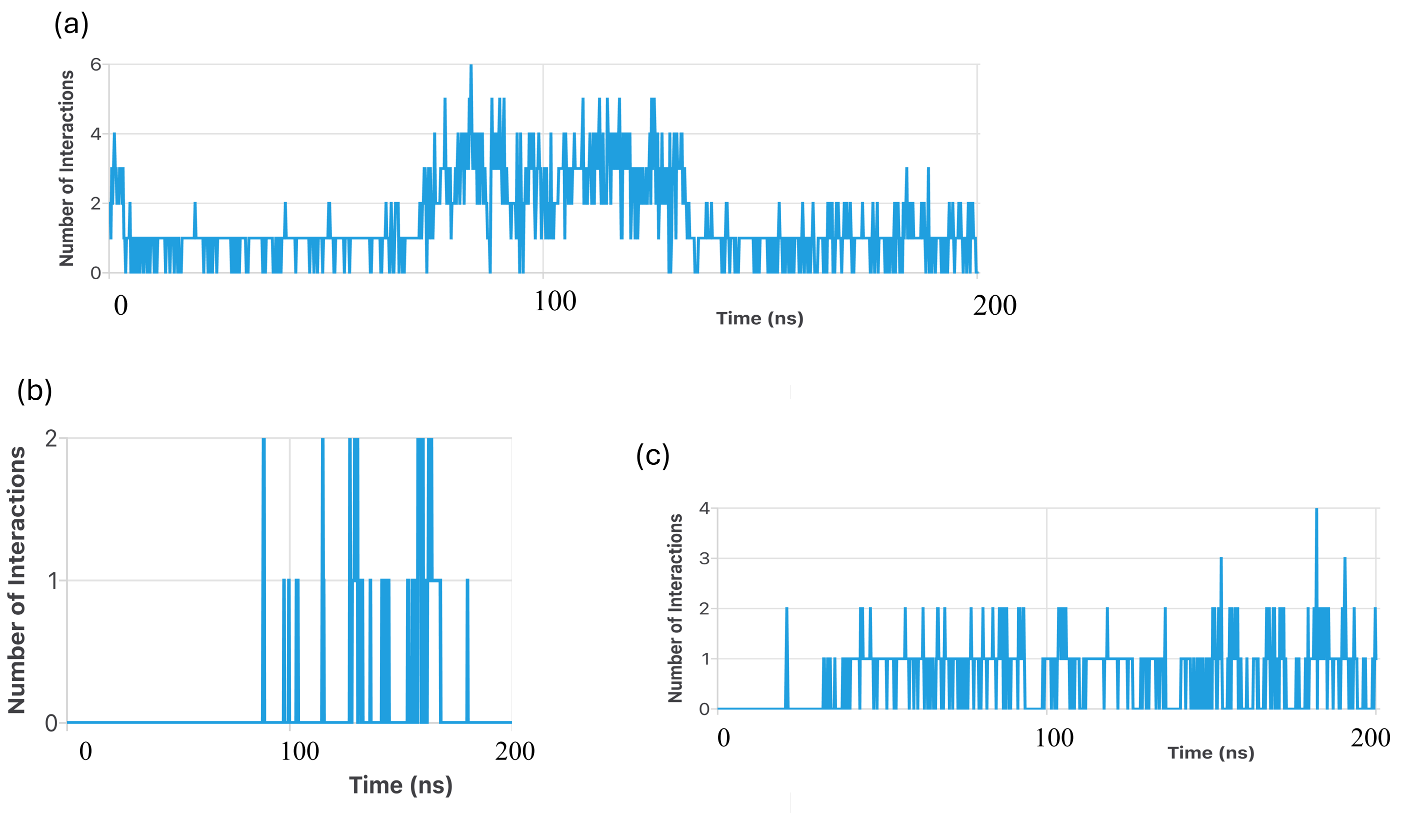

Figure S4. **Time evolution of hydrogen bond interactions between the top-scoring ligand and its corresponding E3 ligase–Aβ42 complex over a 100 ns molecular dynamics simulation**.
(a) VHL-ligand complex, (b) MDM2-ligand complex, and (c) CRBN-ligand complex. The y-axis indicates the number of hydrogen bonds formed between the ligand and protein throughout the simulation, while the x-axis shows the simulation time in nanoseconds (ns). These interaction profiles provide insights into the stability and persistence of ligand binding within the ternary complex interface

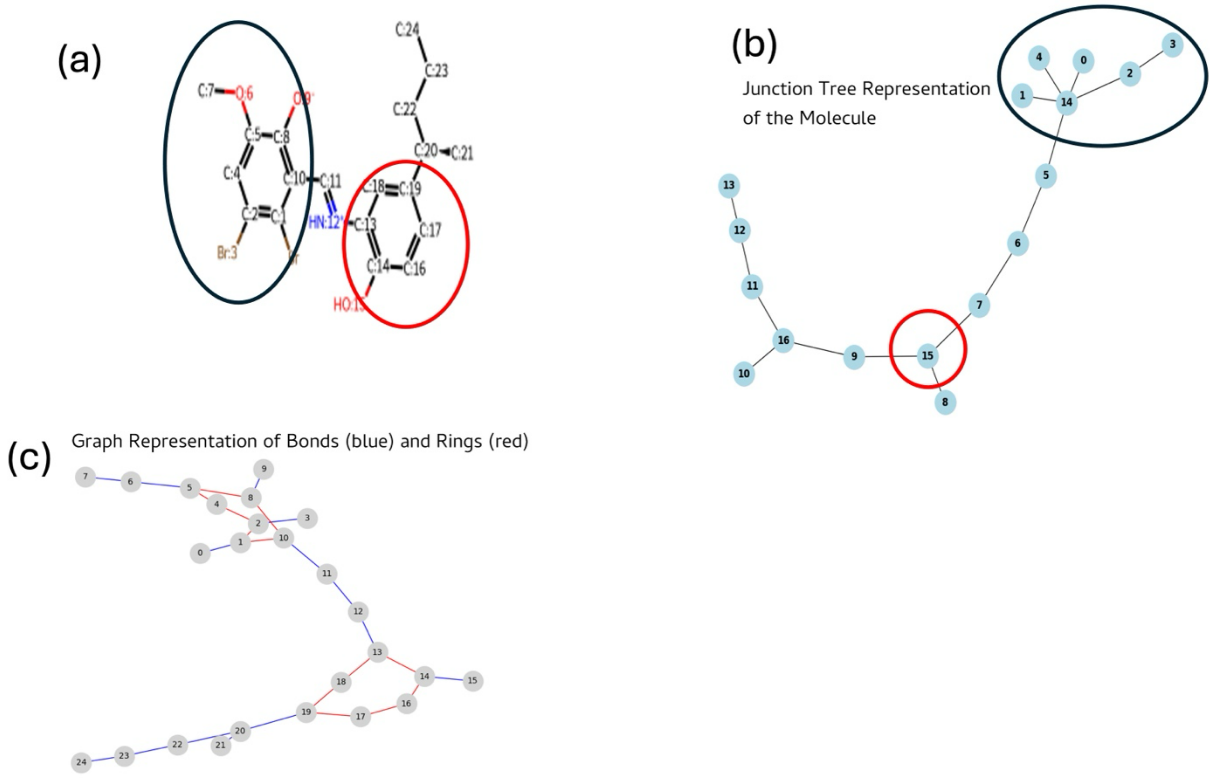

Figure S5. **Graph and Junction Tree representations of a molecule used for JTNN-VAE training.** (**a**) Original molecular structure annotated with atom indices. Two representative substructures (cliques) are highlighted: a brominated aromatic ring (black circle) and a hydroxyl-substituted phenyl ring (red circle). (**b)** Junction tree representation of the molecule, where each node corresponds to a chemically valid clique (e.g., ring, functional group, or bridged system). The circled nodes (14 and 15) represent the cliques highlighted in the molecular structure. Edges represent connectivity based on shared atoms between cliques. (**c**) Molecular graph representation, where atoms are nodes and bonds are edges. Blue edges represent standard bonds, while red edges denote bonds participating in ring structures.

Table S1. List of cliques used in the junction tree, with each bracket representing a set of atom indices forming a maximal subgraph (clique). Indices match the atom numbers from the original molecule and the junction tree nodes.

Indices	Cliques
0	[0, 1]
1	[2, 3]
2	[5, 6]

3	[6, 7]
4	[8, 9]
5	[10, 11]
6	[11, 12]
7	[12, 13]
8	[14, 15]
9	[19, 20]
10	[20, 21]
11	[22, 23]
12	[23, 24]
13	[1, 10, 8, 5, 4, 2]
14	[14, 16, 17, 19, 18, 13]

Table S2. The 50 exemplar molecular compounds showing the SMILES string representation along with its corresponding junction tree structure, which includes cliques (clusters of atoms forming subgraphs) and edges (connections between cliques).

Smiles	Cliques	Edges
Brc1c(Br)cc(OC)c([O-])c1/C=[NH+]/c(c(O)cc2)cc2[C@H](C)CCC	[[0, 1], [2, 3], [5, 6], [6, 7], [8, 9], [10, 11], [11, 12], [12, 13], [14, 15], [19, 20], [20, 21], [20, 22], [22, 23], [23, 24], [1, 10, 8, 5, 4, 2], [14, 16, 17, 19, 18, 13], [20]]	[(0, 14), (1, 14), (2, 3), (2, 14), (4, 14), (5, 6), (5, 14), (6, 7), (7, 15), (8, 15), (9, 15), (9, 16), (10, 16), (11, 12), (11, 16), (12, 13)]
Brc1cc(C)cc(Br)c1OCC(=O)N\N=C\c2c(O)c(O)c(O)cc2	[[0, 1], [3, 4], [6, 7], [8, 9], [9, 10], [10, 11], [11, 12], [11, 13], [13, 14], [14, 15], [15, 16],	[(0, 14), (1, 14), (2, 14), (3, 4), (3, 14), (4, 5), (5, 16), (6, 16), (7, 8), (7, 16), (8, 9), (9, 10),

	[17, 18], [19, 20], [21, 22], [1, 8, 6, 5, 3, 2], [17, 19, 21, 23, 24, 16], [11]]	(10, 15), (11, 15), (12, 15), (13, 15)]
Brc1ccc(nc1)NC(=O)CSc([nH]c2=O)[nH+]c([O-])c2-c3ccccc3	[[0, 1], [4, 7], [7, 8], [8, 9], [8, 10], [10, 11], [11, 12], [14, 15], [17, 18], [19, 20], [1, 6, 5, 4, 3, 2], [13, 14, 19, 17, 16, 12], [21, 22, 23, 24, 25, 20], [8]]	[(0, 10), (1, 2), (1, 10), (2, 13), (3, 13), (4, 5), (4, 13), (5, 6), (6, 11), (7, 11), (8, 11), (9, 11), (9, 12)]
Brc1cccc(O)c1/C=N/c(c2)ccc(c23)[nH+]c(C)[nH]3	[[0, 1], [5, 6], [7, 8], [8, 9], [9, 10], [17, 18], [1, 7, 5, 4, 3, 2], [11, 15, 14, 13, 12, 10], [16, 14, 15, 19, 17]]	[(0, 6), (1, 6), (2, 3), (2, 6), (3, 4), (4, 7), (5, 8), (7, 8)]
Brc1cccc([O-])c1/C=N/c(c2)ccc(c23)[nH+]c(C)[nH]3	[[0, 1], [5, 6], [7, 8], [8, 9], [9, 10], [17, 18], [1, 7, 5, 4, 3, 2], [11, 15, 14, 13, 12, 10], [16, 14, 15, 19, 17]]	[(0, 6), (1, 6), (2, 3), (2, 6), (3, 4), (4, 7), (5, 8), (7, 8)]
C#CC(C)(C)[NH2+]CCC(=O)C1=CCCCC1	[[0, 1], [1, 2], [2, 3], [2, 4], [2, 5], [5, 6], [6, 7], [7, 8], [8, 9], [8, 10], [10, 15, 14, 13, 12, 11], [2], [8]]	[(0, 1), (1, 11), (2, 11), (3, 11), (4, 5), (4, 11), (5, 6), (6, 7), (7, 12), (8, 12), (9, 10), (9, 12)]
C#CC[N@@H+](C(C)(C)C)CC(=O)c1ccccc1	[[0, 1], [1, 2], [2, 3], [3, 4], [4, 5], [4, 6], [4, 7], [3, 8], [8, 9], [9, 10], [9, 11], [11, 16, 15, 14, 13, 12], [3], [4], [9]]	[(0, 1), (1, 2), (2, 12), (3, 12), (3, 13), (4, 13), (5, 13), (6, 13), (7, 8), (7, 12), (8, 14), (9, 14), (10, 11), (10, 14)]
C1=CC=C[C@@H]([C@H]12)NC=C2[C@@]3(C(=O)Nc(c34)cccc4)c5c[nH]c(c56)cccc6	[[8, 9], [10, 11], [9, 19], [0, 5, 4, 3, 2, 1], [6, 4, 5, 8, 7], [10, 9, 14, 13, 12], [15, 16, 17, 18, 14, 13], [20, 19, 23, 22, 21], [24, 25, 26, 27, 23, 22], [9]]	[(0, 4), (0, 9), (1, 5), (2, 7), (2, 9), (3, 4), (5, 6), (5, 9), (7, 8)]
C1=CC=C[C@H]([C@@H]12)NC=C2[C@@]3(C(=O)Nc(c34)cccc4)c5c[nH]c(c56)cccc6	[[8, 9], [10, 11], [9, 19], [0, 5, 4, 3, 2, 1], [6, 4, 5, 8, 7], [10, 9, 14, 13, 12], [15, 16, 17, 18, 14, 13], [20, 19, 23, 22, 21], [24, 25, 26, 27, 23, 22], [9]]	[(0, 4), (0, 9), (1, 5), (2, 7), (2, 9), (3, 4), (5, 6), (5, 9), (7, 8)]
C1=CC=C[C@H]([C@@H]12)NC=C2[C@]3(C(=O)Nc(c34)cccc4)c5c[nH]c(c56)cccc6	[[8, 9], [10, 11], [9, 19], [0, 5, 4, 3, 2, 1], [6, 4, 5, 8, 7], [10, 9, 14, 13, 12], [15, 16, 17, 18, 14, 13], [20, 19, 23, 22, 21], [24, 25, 26, 27, 23, 22], [9]]	[(0, 4), (0, 9), (1, 5), (2, 7), (2, 9), (3, 4), (5, 6), (5, 9), (7, 8)]
C1CC1C[N@@H+](CCC)[C@@H](C2=O)CC(=O)N2c3ccccc3	[[2, 3], [3, 4], [4, 5], [5, 6], [6, 7], [4, 8], [9, 10], [12, 13], [14, 15], [0, 1, 2], [9, 8, 11, 12, 14], [16, 17, 18, 19, 20, 15], [4]]	[(0, 1), (0, 9), (1, 12), (2, 3), (2, 12), (3, 4), (5, 10), (5, 12), (6, 10), (7, 10), (8, 10), (8, 11)]
C1CC1C[N@@H+](CCC)[C@H](C2=O)CC(=O)N2c3ccccc3	[[2, 3], [3, 4], [4, 5], [5, 6], [6, 7], [4, 8], [9, 10], [12, 13], [14, 15], [0, 1, 2], [9, 8, 11, 12, 14], [16, 17, 18, 19, 20, 15], [4]]	[(0, 1), (0, 9), (1, 12), (2, 3), (2, 12), (3, 4), (5, 10), (5, 12), (6, 10), (7, 10), (8, 10), (8, 11)]
C1CC1C[N@H+](CCC)[C@@H](C2=O)CC(=O)N2c3ccccc3	[[2, 3], [3, 4], [4, 5], [5, 6], [6, 7], [4, 8], [9, 10], [12, 13], [14, 15], [0, 1, 2], [9, 8, 11, 12, 14], [16, 17, 18, 19, 20, 15], [4]]	[(0, 1), (0, 9), (1, 12), (2, 3), (2, 12), (3, 4), (5, 10), (5, 12), (6, 10), (7, 10), (8, 10), (8, 11)]
C1CC1C[N@H+](CCC)[C@H](C2=O)CC(=O)N2c3ccccc3	[[2, 3], [3, 4], [4, 5], [5, 6], [6, 7], [4, 8], [9, 10], [12, 13], [14, 15], [0, 1, 2], [9, 8, 11, 12, 14], [16, 17, 18, 19, 20, 15], [4]]	[(0, 1), (0, 9), (1, 12), (2, 3), (2, 12), (3, 4), (5, 10), (5, 12), (6, 10), (7, 10), (8, 10), (8, 11)]

C1CC2CCN1[C@@H]([C@@]23O)[C@@H](NO3)c4c(O)cccc4	[[7, 8], [9, 12], [13, 14], [10, 9, 6, 7, 11], [0, 1, 2, 3, 4, 5, 6, 7], [13, 15, 16, 17, 18, 12]]	[(0, 3), (1, 3), (1, 5), (2, 5), (3, 4)]
C1CC2CCN1[C@@H]([C@@]23O)[C@H](NO3)c4c(O)cccc4	[[7, 8], [9, 12], [13, 14], [10, 9, 6, 7, 11], [0, 1, 2, 3, 4, 5, 6, 7], [13, 15, 16, 17, 18, 12]]	[(0, 3), (1, 3), (1, 5), (2, 5), (3, 4)]
C1CC2CCN1[C@@H]([C@]23O)[C@H](NO3)c4c(O)cccc4	[[7, 8], [9, 12], [13, 14], [10, 9, 6, 7, 11], [0, 1, 2, 3, 4, 5, 6, 7], [13, 15, 16, 17, 18, 12]]	[(0, 3), (1, 3), (1, 5), (2, 5), (3, 4)]
C1CCCC1[NH2+]Cc2cc(O)c(cc2)OC	[[4, 5], [5, 6], [6, 7], [9, 10], [11, 14], [14, 15], [0, 1, 2, 3, 4], [8, 9, 11, 12, 13, 7]]	[(0, 1), (0, 6), (1, 2), (2, 7), (3, 7), (4, 5), (4, 7)]
C1CCCCC12C(=C)N(C(=O)O2)C[C@@H]3[NH2+]CCc(c34)cccc4	[[6, 7], [9, 10], [8, 12], [12, 13], [0, 5, 4, 3, 2, 1], [6, 5, 11, 9, 8], [14, 15, 16, 17, 18, 13], [19, 20, 21, 22, 18, 17]]	[(0, 5), (1, 5), (2, 3), (2, 5), (3, 6), (4, 5), (6, 7)]
C1CCCCC12C(C#N)=C3N(C(=O)[C@H]2C#N)[C@@]4([C@H](S3)CCCC4)OCC(=O)c5ccccc5	[[6, 7], [7, 8], [11, 12], [13, 14], [14, 15], [16, 23], [23, 24], [24, 25], [25, 26], [25, 27], [0, 5, 4, 3, 2, 1], [6, 9, 10, 11, 13, 5], [18, 17, 16, 10, 9], [19, 20, 21, 22, 16, 17], [28, 29, 30, 31, 32, 27], [25]]	[(0, 1), (0, 11), (2, 11), (3, 4), (3, 11), (5, 6), (5, 12), (6, 7), (7, 15), (8, 15), (9, 14), (9, 15), (10, 11), (11, 12), (12, 13)]
C1CCCCC1OC[C@@H](O)CN2CC[NH+](CC2)C[C@@H](O)COC3CCCCC3	[[5, 6], [6, 7], [7, 8], [8, 9], [8, 10], [10, 11], [14, 17], [17, 18], [18, 19], [18, 20], [20, 21], [21, 22], [0, 5, 4, 3, 2, 1], [12, 13, 14, 15, 16, 11], [23, 24, 25, 26, 27, 22], [8], [18]]	[(0, 1), (0, 12), (1, 2), (2, 15), (3, 15), (4, 5), (4, 15), (5, 13), (6, 7), (6, 13), (7, 16), (8, 16), (9, 10), (9, 16), (10, 11), (11, 14)]
C1CCCCC1OC[C@@H](O)CN2CC[NH+](CC2)C[C@H](O)COC3CCCCC3	[[5, 6], [6, 7], [7, 8], [8, 9], [8, 10], [10, 11], [14, 17], [17, 18], [18, 19], [18, 20], [20, 21], [21, 22], [0, 5, 4, 3, 2, 1], [12, 13, 14, 15, 16, 11], [23, 24, 25, 26, 27, 22], [8], [18]]	[(0, 1), (0, 12), (1, 2), (2, 15), (3, 15), (4, 5), (4, 15), (5, 13), (6, 7), (6, 13), (7, 16), (8, 16), (9, 10), (9, 16), (10, 11), (11, 14)]
C1CCCCC1OC[C@@H](O)C[N@H+]2CC[N@@H+](CC2)C[C@H](O)COC3CCCCC3	[[5, 6], [6, 7], [7, 8], [8, 9], [8, 10], [10, 11], [14, 17], [17, 18], [18, 19], [18, 20], [20, 21], [21, 22], [0, 5, 4, 3, 2, 1], [12, 13, 14, 15, 16, 11], [23, 24, 25, 26, 27, 22], [8], [18]]	[(0, 1), (0, 12), (1, 2), (2, 15), (3, 15), (4, 5), (4, 15), (5, 13), (6, 7), (6, 13), (7, 16), (8, 16), (9, 10), (9, 16), (10, 11), (11, 14)]
C1CCCCC1OC[C@@H](O)C[N@H+]2CC[N@H+](CC2)C[C@H](O)COC3CCCCC3	[[5, 6], [6, 7], [7, 8], [8, 9], [8, 10], [10, 11], [14, 17], [17, 18], [18, 19], [18, 20], [20, 21], [21, 22], [0, 5, 4, 3, 2, 1], [12, 13, 14, 15, 16, 11], [23, 24, 25, 26, 27, 22], [8], [18]]	[(0, 1), (0, 12), (1, 2), (2, 15), (3, 15), (4, 5), (4, 15), (5, 13), (6, 7), (6, 13), (7, 16), (8, 16), (9, 10), (9, 16), (10, 11), (11, 14)]
C1CCCCC1OC[C@H](O)CN2CC[NH+](CC2)C[C@@H](O)COC3CCCCC3	[[5, 6], [6, 7], [7, 8], [8, 9], [8, 10], [10, 11], [14, 17], [17, 18], [18, 19], [18, 20], [20, 21], [21, 22], [0, 5, 4, 3, 2, 1], [12, 13, 14, 15, 16, 11], [23, 24, 25, 26, 27, 22], [8], [18]]	[(0, 1), (0, 12), (1, 2), (2, 15), (3, 15), (4, 5), (4, 15), (5, 13), (6, 7), (6, 13), (7, 16), (8, 16), (9, 10), (9, 16), (10, 11), (11, 14)]
C1CCCCC1OC[C@H](O)CN2CC[NH+](C	[[5, 6], [6, 7], [7, 8], [8, 9], [8, 10], [10, 11], [14, 17], [17, 18], [18, 19], [18, 20], [20, 21],	[(0, 1), (0, 12), (1, 2), (2, 15), (3, 15), (4, 5), (4, 15), (5, 13), (6,

C2)C[C@H](O)COC3CCCCC3	[21, 22], [0, 5, 4, 3, 2, 1], [12, 13, 14, 15, 16, 11], [23, 24, 25, 26, 27, 22], [8], [18]]	7), (6, 13), (7, 16), (8, 16), (9, 10), (9, 16), (10, 11), (11, 14)]
C1CCCCC1OC[C@H](O)C[N@H+]2CC[N@@H+](CC2)C[C@@H](O)COC3CCCCC3	[[5, 6], [6, 7], [7, 8], [8, 9], [8, 10], [10, 11], [14, 17], [17, 18], [18, 19], [18, 20], [20, 21], [21, 22], [0, 5, 4, 3, 2, 1], [12, 13, 14, 15, 16, 11], [23, 24, 25, 26, 27, 22], [8], [18]]	[(0, 1), (0, 12), (1, 2), (2, 15), (3, 15), (4, 5), (4, 15), (5, 13), (6, 7), (6, 13), (7, 16), (8, 16), (9, 10), (9, 16), (10, 11), (11, 14)]
C1CCCCC1OC[C@H](O)C[N@H+]2CC[N@@H+](CC2)C[C@H](O)COC3CCCCC3	[[5, 6], [6, 7], [7, 8], [8, 9], [8, 10], [10, 11], [14, 17], [17, 18], [18, 19], [18, 20], [20, 21], [21, 22], [0, 5, 4, 3, 2, 1], [12, 13, 14, 15, 16, 11], [23, 24, 25, 26, 27, 22], [8], [18]]	[(0, 1), (0, 12), (1, 2), (2, 15), (3, 15), (4, 5), (4, 15), (5, 13), (6, 7), (6, 13), (7, 16), (8, 16), (9, 10), (9, 16), (10, 11), (11, 14)]
C1CCCCC1OC[C@H](O)C[N@H+]2CC[N@H+](CC2)C[C@@H](O)COC3CCCCC3	[[5, 6], [6, 7], [7, 8], [8, 9], [8, 10], [10, 11], [14, 17], [17, 18], [18, 19], [18, 20], [20, 21], [21, 22], [0, 5, 4, 3, 2, 1], [12, 13, 14, 15, 16, 11], [23, 24, 25, 26, 27, 22], [8], [18]]	[(0, 1), (0, 12), (1, 2), (2, 15), (3, 15), (4, 5), (4, 15), (5, 13), (6, 7), (6, 13), (7, 16), (8, 16), (9, 10), (9, 16), (10, 11), (11, 14)]
C1CCCCC1OC[C@H](O)C[N@H+]2CC[N@H+](CC2)C[C@H](O)COC3CCCCC3	[[5, 6], [6, 7], [7, 8], [8, 9], [8, 10], [10, 11], [14, 17], [17, 18], [18, 19], [18, 20], [20, 21], [21, 22], [0, 5, 4, 3, 2, 1], [12, 13, 14, 15, 16, 11], [23, 24, 25, 26, 27, 22], [8], [18]]	[(0, 1), (0, 12), (1, 2), (2, 15), (3, 15), (4, 5), (4, 15), (5, 13), (6, 7), (6, 13), (7, 16), (8, 16), (9, 10), (9, 16), (10, 11), (11, 14)]
C1CCCCC1[N@H+](C)Cc2c(O)ccc(c23)cccc3	[[5, 6], [6, 7], [6, 8], [8, 9], [10, 11], [0, 5, 4, 3, 2, 1], [10, 12, 13, 14, 15, 9], [16, 17, 18, 19, 15, 14], [6]]	[(0, 5), (0, 8), (1, 8), (2, 3), (2, 8), (3, 6), (4, 6), (6, 7)]
C1CCCCN1C(=O)c2ccc(cc2)\N=C\c3c(O)c(O)c(O)cc3	[[5, 6], [6, 7], [6, 8], [11, 14], [14, 15], [15, 16], [17, 18], [19, 20], [21, 22], [0, 5, 4, 3, 2, 1], [9, 10, 11, 12, 13, 8], [17, 19, 21, 23, 24, 16], [6]]	[(0, 9), (0, 12), (1, 12), (2, 10), (2, 12), (3, 4), (3, 10), (4, 5), (5, 11), (6, 11), (7, 11), (8, 11)]
C1CCCCN1C(=O)c2ccc(cc2)\[NH+]=C\c3c(O)c(O)c(O)cc3	[[5, 6], [6, 7], [6, 8], [11, 14], [14, 15], [15, 16], [17, 18], [19, 20], [21, 22], [0, 5, 4, 3, 2, 1], [9, 10, 11, 12, 13, 8], [17, 19, 21, 23, 24, 16], [6]]	[(0, 9), (0, 12), (1, 12), (2, 10), (2, 12), (3, 4), (3, 10), (4, 5), (5, 11), (6, 11), (7, 11), (8, 11)]
C1CCCCN1C(=O)c2ccc(cc2)\[NH+]=C\c3c([O-])cc(O)cc3	[[5, 6], [6, 7], [6, 8], [11, 14], [14, 15], [15, 16], [17, 18], [20, 21], [0, 5, 4, 3, 2, 1], [9, 10, 11, 12, 13, 8], [17, 19, 20, 22, 23, 16], [6]]	[(0, 8), (0, 11), (1, 11), (2, 9), (2, 11), (3, 4), (3, 9), (4, 5), (5, 10), (6, 10), (7, 10)]
C1CCCCN1CCN(C2=O)c(cccc3)c3[C@@]24NN=C(S4)c(cc5)ccc5C	[[5, 6], [6, 7], [7, 8], [9, 10], [20, 22], [27, 28], [0, 5, 4, 3, 2, 1], [9, 8, 11, 16, 17], [12, 13, 14, 15, 16, 11], [18, 17, 21, 20, 19], [23, 24, 27, 26, 25, 22]]	[(0, 1), (0, 6), (1, 2), (2, 7), (3, 7), (4, 9), (4, 10), (5, 10), (7, 8), (7, 9)]
C1CCCCN1Cc([nH+]c(c23)cccc2)n3Cc4ccc(Br)cc4	[[5, 6], [6, 7], [15, 16], [16, 17], [20, 21], [0, 5, 4, 3, 2, 1], [8, 7, 15, 10, 9], [11, 12, 13, 14, 10, 9], [18, 19, 20, 22, 23, 17]]	[(0, 1), (0, 5), (1, 6), (2, 3), (2, 6), (3, 8), (4, 8), (6, 7)]
C1CCCCN1Cc(n2)n(c(c23)cccc3)Cc4ccc(Br)cc4	[[5, 6], [6, 7], [9, 16], [16, 17], [20, 21], [0, 5, 4, 3, 2, 1], [8, 7, 9, 10, 11], [12, 13, 14, 15, 11, 10], [18, 19, 20, 22, 23, 17]]	[(0, 1), (0, 5), (1, 6), (2, 3), (2, 6), (3, 8), (4, 8), (6, 7)]
C1CCCCN1[C@@H](C)c([nH+]c(c23)cccc2)n3CCOc4cc(OC)ccc4	[[5, 6], [6, 7], [6, 8], [16, 17], [17, 18], [18, 19], [19, 20], [22, 23], [23, 24], [0, 5, 4, 3, 2, 1], [9, 8, 16, 11, 10], [12, 13, 14, 15, 11, 10], [21, 22, 25, 26, 27, 20], [6]]	[(0, 9), (0, 13), (1, 13), (2, 10), (2, 13), (3, 4), (3, 10), (4, 5), (5, 6), (6, 12), (7, 8), (7, 12), (10, 11)]
C1CCCCN1[C@]2(C(=O)C)CC[N@@H+]	[[5, 6], [6, 7], [7, 8], [7, 9], [12, 15], [16, 17], [19, 20], [21, 22], [22, 23], [26, 27], [0, 5, 4, 3,	[(0, 10), (0, 14), (1, 14), (1, 15), (2, 15), (3, 15), (4, 11), (4, 12),

(CC2)[C@@H](C3=O)CC(=O)N3Cc4ccc(Cl)cc4	2, 1], [10, 11, 12, 13, 14, 6], [16, 15, 18, 19, 21], [24, 25, 26, 28, 29, 23], [6], [7]]	(5, 12), (6, 12), (7, 8), (7, 12), (8, 13), (9, 13), (11, 14)]
C1CCCCN1[C@]2(C(=O)C)CC[N@H+](CC2)[C@H](C3=O)CC(=O)N3Cc4ccc(Cl)cc4	[[5, 6], [6, 7], [7, 8], [7, 9], [12, 15], [16, 17], [19, 20], [21, 22], [22, 23], [26, 27], [0, 5, 4, 3, 2, 1], [10, 11, 12, 13, 14, 6], [16, 15, 18, 19, 21], [24, 25, 26, 28, 29, 23], [6], [7]]	[(0, 10), (0, 14), (1, 14), (1, 15), (2, 15), (3, 15), (4, 11), (4, 12), (5, 12), (6, 12), (7, 8), (7, 12), (8, 13), (9, 13), (11, 14)]
C1CCCCN1[C@]2(C(=O)N)CCN(CC2)[C@H](C3=O)CC(=O)N3CC(c4ccccc4)c5ccccc5	[[5, 6], [6, 7], [7, 8], [7, 9], [12, 15], [16, 17], [19, 20], [21, 22], [22, 23], [23, 24], [23, 30], [0, 5, 4, 3, 2, 1], [10, 11, 12, 13, 14, 6], [16, 15, 18, 19, 21], [25, 26, 27, 28, 29, 24], [31, 32, 33, 34, 35, 30], [6], [7], [23]]	[(0, 11), (0, 16), (1, 16), (1, 17), (2, 17), (3, 17), (4, 12), (4, 13), (5, 13), (6, 13), (7, 8), (7, 13), (8, 18), (9, 14), (9, 18), (10, 15), (10, 18), (12, 16)]
C1CCCCN1c2nc(nc(n2)OCC(F)(F)F)N3CC[NH+](CC3)Cc4ccccc4	[[5, 6], [10, 12], [12, 13], [13, 14], [14, 15], [14, 16], [14, 17], [8, 18], [21, 24], [24, 25], [0, 5, 4, 3, 2, 1], [7, 8, 9, 10, 11, 6], [19, 20, 21, 22, 23, 18], [26, 27, 28, 29, 30, 25], [14]]	[(0, 10), (0, 11), (1, 2), (1, 11), (2, 3), (3, 14), (4, 14), (5, 14), (6, 14), (7, 11), (7, 12), (8, 9), (8, 12), (9, 13)]
C1CCCC[NH+]1C2(C(=O)C)CCN(CC2)[C@@H](C3=O)CC(=O)N3Cc4ccc(Cl)cc4	[[5, 6], [6, 7], [7, 8], [7, 9], [12, 15], [16, 17], [19, 20], [21, 22], [22, 23], [26, 27], [0, 5, 4, 3, 2, 1], [10, 11, 12, 13, 14, 6], [16, 15, 18, 19, 21], [24, 25, 26, 28, 29, 23], [6], [7]]	[(0, 10), (0, 14), (1, 14), (1, 15), (2, 15), (3, 15), (4, 11), (4, 12), (5, 12), (6, 12), (7, 8), (7, 12), (8, 13), (9, 13), (11, 14)]
C1CCCC[NH+]1C2(C(=O)C)CCN(CC2)[C@H](C3=O)CC(=O)N3Cc4ccc(Cl)cc4	[[5, 6], [6, 7], [7, 8], [7, 9], [12, 15], [16, 17], [19, 20], [21, 22], [22, 23], [26, 27], [0, 5, 4, 3, 2, 1], [10, 11, 12, 13, 14, 6], [16, 15, 18, 19, 21], [24, 25, 26, 28, 29, 23], [6], [7]]	[(0, 10), (0, 14), (1, 14), (1, 15), (2, 15), (3, 15), (4, 11), (4, 12), (5, 12), (6, 12), (7, 8), (7, 12), (8, 13), (9, 13), (11, 14)]
C1CCCC[NH+]1C2(C(=O)N)CCN(CC2)[C@H](C3=O)CC(=O)N3CC(c4ccccc4)c5ccccc5	[[5, 6], [6, 7], [7, 8], [7, 9], [12, 15], [16, 17], [19, 20], [21, 22], [22, 23], [23, 24], [23, 30], [0, 5, 4, 3, 2, 1], [10, 11, 12, 13, 14, 6], [16, 15, 18, 19, 21], [25, 26, 27, 28, 29, 24], [31, 32, 33, 34, 35, 30], [6], [7], [23]]	[(0, 11), (0, 16), (1, 16), (1, 17), (2, 17), (3, 17), (4, 12), (4, 13), (5, 13), (6, 13), (7, 8), (7, 13), (8, 18), (9, 14), (9, 18), (10, 15), (10, 18), (12, 16)]
C1CCCC[NH+]1CC(=O)N\N=C\c2c(cccc2)OCc3ccccc3	[[5, 6], [6, 7], [7, 8], [7, 9], [9, 10], [10, 11], [11, 12], [13, 18], [18, 19], [19, 20], [0, 5, 4, 3, 2, 1], [14, 15, 16, 17, 12, 13], [21, 22, 23, 24, 25, 20], [7]]	[(0, 1), (0, 10), (1, 13), (2, 13), (3, 4), (3, 13), (4, 5), (5, 6), (6, 11), (7, 8), (7, 11), (8, 9), (9, 12)]
C1CCCC[NH+]1CC(=O)N\N=C\c2ccc(cc2)N(C)C	[[5, 6], [6, 7], [7, 8], [7, 9], [9, 10], [10, 11], [11, 12], [15, 18], [18, 19], [18, 20], [0, 5, 4, 3, 2, 1], [13, 14, 15, 16, 17, 12], [7], [18]]	[(0, 1), (0, 10), (1, 12), (2, 12), (3, 4), (3, 12), (4, 5), (5, 6), (6, 11), (7, 11), (7, 13), (8, 13), (9, 13)]
C1CCCC[NH+]1CC(=O)Nc(c(C)c(s2)C)c2C(=O)OCC	[[5, 6], [6, 7], [7, 8], [7, 9], [9, 10], [11, 12], [13, 15], [16, 17], [17, 18], [17, 19], [19, 20], [20, 21], [0, 5, 4, 3, 2, 1], [11, 10, 16, 14, 13], [7], [17]]	[(0, 1), (0, 12), (1, 14), (2, 14), (3, 4), (3, 14), (4, 13), (5, 13), (6, 13), (7, 13), (7, 15), (8, 15), (9, 10), (9, 15), (10, 11)]
C1CCCC[NH+]1CC(=O)Nc(cc2)ccc2C(=O)N(CC)CC	[[5, 6], [6, 7], [7, 8], [7, 9], [9, 10], [15, 16], [16, 17], [16, 18], [18, 19], [19, 20], [18, 21], [21, 22], [0, 5, 4, 3, 2, 1], [11, 12, 15, 14, 13, 10], [7], [16], [18]]	[(0, 1), (0, 12), (1, 14), (2, 14), (3, 4), (3, 14), (4, 13), (5, 13), (5, 15), (6, 15), (7, 15), (7, 16), (8, 9), (8, 16), (10, 11), (10, 16)]

C1CCCC[NH+]1CCC(=O)O[C@@H]2[C@H](CCCC2)S(=O)(=O)CCCC	[[5, 6], [6, 7], [7, 8], [8, 9], [8, 10], [10, 11], [12, 17], [17, 18], [17, 19], [17, 20], [20, 21], [21, 22], [22, 23], [0, 5, 4, 3, 2, 1], [12, 13, 14, 15, 16, 11], [8], [17]]	[(0, 1), (0, 13), (1, 2), (2, 15), (3, 15), (4, 5), (4, 15), (5, 14), (6, 14), (6, 16), (7, 16), (8, 16), (9, 10), (9, 16), (10, 11), (11, 12)]

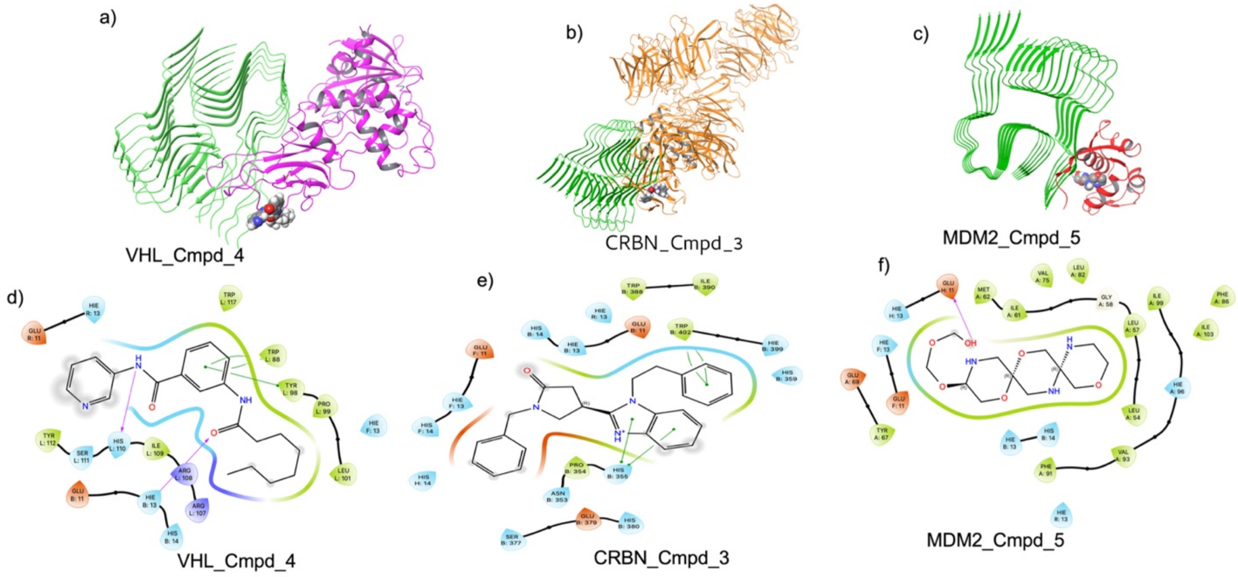

Figure S4. Top most ranked AI-generated compounds docked to E3 ligase–Aβ42 complexes. (a) VHL_Cmpd_4 bound to VHL–Aβ42 complex; (b) CRBN_Cmpd_2 bound to CRBN–Aβ42 complex; (c) MDM2_Cmpd_5 bound to MDM2–Aβ42 complex. (d–f) Corresponding 2D interaction diagrams showing key hydrogen bonds and hydrophobic contacts stabilizing each ligand within the E3 binding sites.

Table S3. Assessment of 3D conformational plausibility of VHL-targeting compounds generated by the Ligase-Conditioned JT-VAE model.

Compounds	Rotatable bonds	RMSD between conformation
VHL_Cmpd_1	13.0	0.000000e+00
VHL_Cmpd_2	3.0	9.262904e-08
VHL_Cmpd_3	11.0	0.000000e+00
VHL_Cmpd_4	7.0	0.000000e+00
VHL_Cmpd_5	10.0	1.348699e-07
VHL_Cmpd_6	1.0	0.000000e+00
VHL_Cmpd_7	4.0	0.000000e+00
VHL_Cmpd_8	12.0	0.000000e+00
VHL_Cmpd_9	8.0	7.446945e-08
VHL_Cmpd_10	3.0	0.000000e+00

Table S4. Assessment of 3D conformational plausibility of CRBN-targeting compounds generated by the Ligase-Conditioned JT-VAE model.

Compounds	Rotatable bonds	RMSD between conformation
CRBN_Cmpd_1	4	0.000000e+00
CRBN_Cmpd_2	6	0.000000e+00
CRBN_Cmpd_3	7	0.000000e+00
CRBN_Cmpd_4	2	0.000000e+00
CRBN_Cmpd_5	9	7.839155e-08
CRBN_Cmpd_6	6	0.000000e+00
CRBN_Cmpd_7	3	5.869460e-08
CRBN_Cmpd_8	2	0.000000e+00
CRBN_Cmpd_9	4	0.000000e+00

Table S5. Assessment of 3D conformational plausibility of MDM2-targeting compounds generated by the Ligase-Conditioned JT-VAE model.

Compounds	Rotatable bonds	RMSD between conformation
MDM2_Cmpd_1	6	0.000000e+00
MDM2_Cmpd_2	11	1.252236e-07
MDM2_Cmpd_3	5	0.000000e+00
MDM2_Cmpd_4	3	0.000000e+00
MDM2_Cmpd_5	6	0.000000e+00
MDM2_Cmpd_6	22	0.000000e+00
MDM2_Cmpd_7	0	0.000000e+00

```
from rdkit import Chem

from rdkit.Chem import AllChem, Descriptors

from rdkit.Chem.rdMolAlign import GetBestRMS

import os

# Define function to evaluate conformer plausibility

def evaluate_conformer_plausibility(smiles):

    mol = Chem.MolFromSmiles(smiles)

    if mol is None:

        return None, "Invalid SMILES"

    mol = Chem.AddHs(mol)

    params = AllChem.ETKDGv3()

    params.randomSeed = 42

    success = AllChem.EmbedMolecule(mol, params)

    if success != 0:

        return None, "Embedding failed"

    AllChem.UFFOptimizeMolecule(mol)

    rot_bonds = Descriptors.NumRotatableBonds(mol)

    mol2 = Chem.AddHs(Chem.MolFromSmiles(smiles))

    AllChem.EmbedMolecule(mol2, params)

    AllChem.UFFOptimizeMolecule(mol2)

    rmsd = GetBestRMS(mol, mol2)

    return {

        "rotatable_bonds": rot_bonds,

        "embedding_success": True,

        "rmsd_between_conformers": rmsd

    }, None

# Load SMILES from uploaded files
smiles_files = sorted([f for f in os.listdir(input_folder)
if f.endswith('.smi')])
results = []

for file_name in smiles_files:

    file_path = os.path.join(input_folder, file_name)

    with open(file_path, 'r') as file:

        smiles = file.readline().strip()

        result, error =
evaluate_conformer_plausibility(smiles)
```

Figure S6. Python script used to evaluate the 3D conformational plausibility of ligase-specific compounds
